# Correlation Between Internal Carotid Artery Tortuosity and Imaging of Cerebral Small Vessel Disease

**DOI:** 10.3389/fneur.2020.567232

**Published:** 2020-10-22

**Authors:** Yuan-Chang Chen, Xiao-Er Wei, Jing Lu, Rui-Hua Qiao, Xue-Feng Shen, Yue-Hua Li

**Affiliations:** Institute of Diagnostic and Interventional Radiology, Shanghai Jiao Tong University Affiliated Sixth People's Hospital, Shanghai, China

**Keywords:** cerebral small vessel disease, internal carotid artery, artery tortuosity, magnetic resonance imaging, computed tomography angiography

## Abstract

**Background and Purpose:** An association between artery tortuosity and neuroimaging of cerebral small vessel disease (SVD) has been reported, especially in the posterior circulation. However, few studies involved the whole magnetic resonance imaging (MRI) spectrum of SVD in association with anterior circulation arterial tortuosity. This study aimed to investigate the relationship between internal carotid artery (ICA) tortuosity and the neuroimaging of SVD.

**Methods:** Data of 1,264 consecutive patients in whom cerebral vessel diseases were suspected and who underwent both MRI and computed tomography angiography were reviewed from a prospective registry. Internal carotid artery tortuosity was evaluated using the tortuosity index (TI), which was defined as the ratio of the vessel centerline length divided by the straight length. Magnetic resonance imaging was used to assess cerebral microbleeds (CMBs), white matter hyperintensities (WMHs), enlarged perivascular spaces (EPVSs), and lacunes.

**Results:** The TIs of the ICA for patients with and without SVD MRI markers were 1.81 ± 0.42 and 1.72 ± 0.33, respectively (*P* < 0.001). Univariate analysis showed that the ICA TI were positively correlated with each SVD MRI marker (*P* < 0.001), and the correlation coefficients (*r*_*s*_) were 0.57, 0.42, 0.30, and 0.26 for EPVSs, WMHs, CMBs, and lacunes, respectively. The adjusted ORs of the ICA TI were 1.52 (95% CI 1.44–1.60, *P* < 0.001) for EPVS grade 1, 2.05 (95% CI 1.93–2.18, *P* < 0.001) for EPVS grades 2–4, and 1.09 (95% CI 1.03–1.15, *P* = 0.004) for WMH grade 3.

**Conclusions:** The TI of ICA was higher in patients with neuroimaging of SVD. Internal carotid arteries tortuosity was associated with MRI-defined markers of SVD, including EPVS and high-grade WMH, and positively correlated with EPVS severity. Arterial tortuosity might be a risk factor for SVD. This finding may have potential clinical significance for identifying patients with suspected SVD.

## Introduction

Blood vessel tortuosity is a common vessel anomaly affecting a range of vessels, from large arteries to small arterioles, in a number of organ systems. Severe tortuosity may cause a variety of serious symptoms (1). Twisted or tortuous arteries have often been reported in cerebral and internal carotid arteries (ICAs) (1), and the prevalence of tortuosity was significantly higher in the ICA system than in the vertebrobasilar system (2). However, a very different prevalence of ICA tortuosity has been reported (3, 4). The disagreement in prevalence may be due to difference in study cohort and diagnostic technique and lack of diagnostic criteria. Several possible measures of tortuosity have been suggested, but none of them have obtained universal acceptance. Tortuosity index (TI) is commonly used as an index of tortuosity because it was a relatively straightforward approach in the absence of diagnostic criteria for anterior circulation vessel tortuosity (2). TI value can be easily measured without special software. In addition, in clinical applications, these values can be rapidly obtained to evaluate the degree of vascular tortuosity.

Cerebral small vessel disease (SVD) refers to all pathological processes that affect the small vessels of the brain, including small arteries and arterioles, capillaries, and small veins (5). There are six closely correlated features in SVD that can be observed on brain magnetic resonance imaging (MRI), including recent small subcortical infarcts, white matter hyperintensities (WMHs), lacunes, cerebral microbleeds (CMBs), enlarged perivascular spaces (EPVSs), and atrophy (6). An association between artery tortuosity and MR neuroimaging markers of SVD has been reported, especially in the posterior circulation (7–9). However, a few studies involved the whole MRI spectrum of SVD in association with ICA tortuosity, while majority of the SVD lesions occur in the basal ganglia and periventricular region (10).

In our previous study, we found that intracranial artery calcification correlated with the neuroimaging findings of SVD, which suggested a certain relation between large vessel disease and SVD. Based on these findings, we hypothesized that large artery tortuosity was associated with SVD. The present study aimed to investigate the correlation between ICA tortuosity and the neuroimaging markers of SVD using craniocervical artery computed tomography angiography (CTA) and to determine if ICA tortuosity is a risk factor for SVD and whether it can act as a marker of disease severity.

## Materials and Methods

### Study Population/Patients

**Figure 1:**
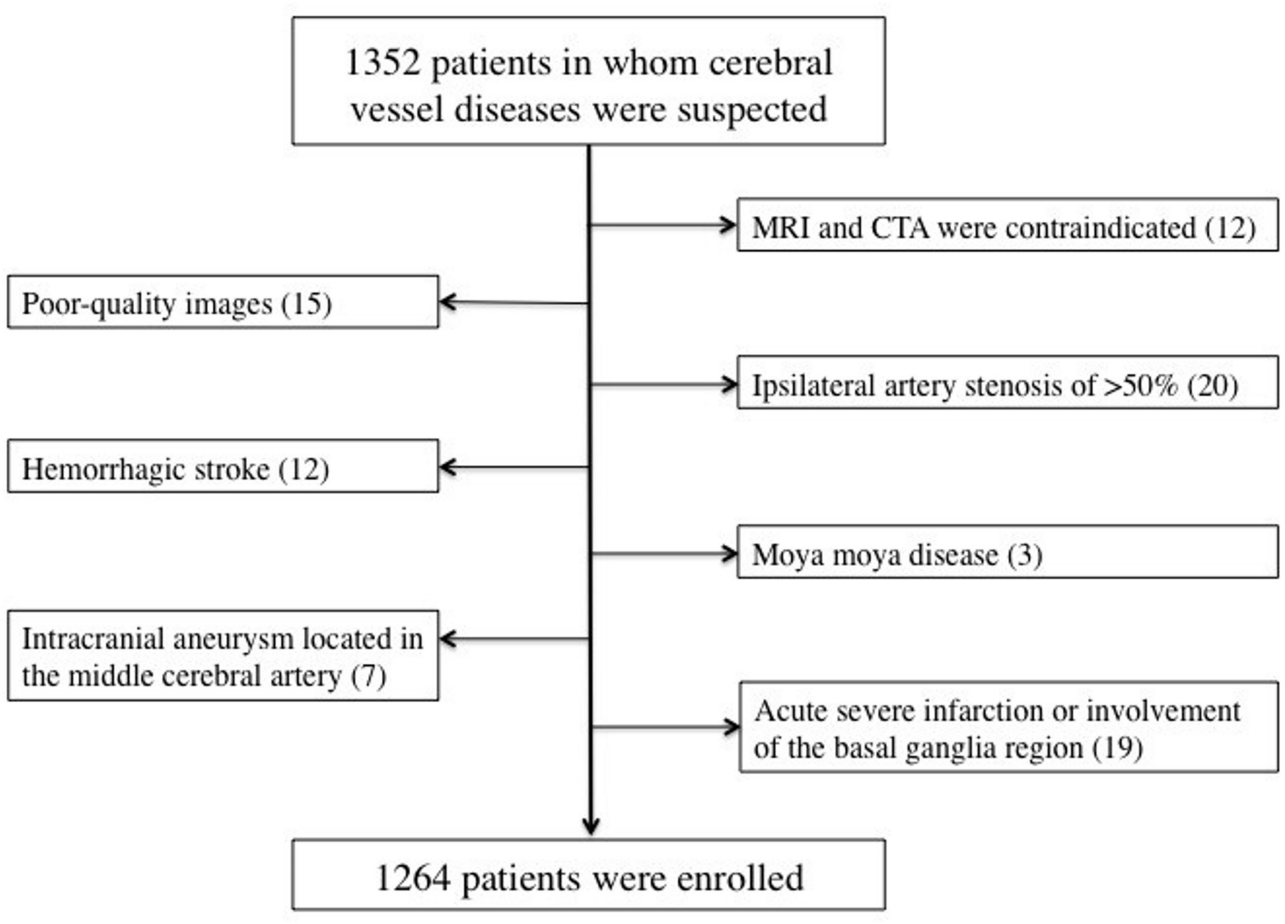
Flowchart of case enrollment.

The present study was a retrospective analysis of prospectively collected registry data for 1,352 consecutive patients who visited our hospital with the onset of a series of symptoms and in whom cerebrovascular disease was suspected by clinical specialists according to these symptoms between July 2018 and December 2019. The inclusion criteria were as follows: (i) patients who had undergone both craniocervical CTA and brain MRI within 7 days of symptom onset; (ii) patients aged between 18 and 90 years; (iii) patients with no critical medical conditions; and (iv) patients with no history of tumors, head trauma, or connective tissue disease. The exclusion criteria were as follows: (i) patients in whom MRI and CTA were contraindicated; (ii) patients in whom poor-quality images were obtained; (iii) patients with ipsilateral artery stenosis of >50%; (iv) patients with acute large area infarction or involvement of the basal ganglia region, which may affect the observation of basal ganglia lesions, or hemorrhagic stroke, or intracranial aneurysm located in the middle cerebral artery that may affect the origin of the penetrating arteries; and (v) patients with other uncommon etiologies such as dissection and moya moya disease. A total of 1,264 patients were included in the study (Figure 1).

This study was carried out in accordance with the recommendations of institutional guidelines. The protocol was approved by the committee of Shanghai Jiao Tong University Affiliated Sixth People's Hospital institutional review board. All subjects provided written informed consent in accordance with the Declaration of Helsinki. This study adhered to standard biosecurity and institutional safety procedures.

### CT Acquisition and Artery Tortuosity Measurement

Computed tomography angiography was performed using a Brilliance 256-channel iCT device (Brilliance iCT, Philips Medical Systems, Haifa, Israel). The parameters were as follows: 150 mA; 120 kVp; pitch, 0.601; slice thickness, 1 mm; and slice acquisition interval, 0.5 mm. An iodinated contrast medium (Iobrix) was intravenously administered (120 ml) at a rate of 3.5–4.0 ml/s by using the autoinjection program.

Computed tomography angiography data were post-processed by using the Vessel Analysis function on a three-dimensional (3D) workstation (Intellispace portal, Philips Medical Systems, Haifa, Israel) to measure centerline vessel length of the bilateral ICAs, beginning from the origin of ICA to the bifurcation of anterior cerebral artery and middle cerebral artery. Then, 3D image reconstruction was performed using maximum intensity projection and volume rendering. The straight length (3D) of ICA was measured on the anteroposterior position. The severity of ICA tortuosity was evaluated using the tortuosity index (TI). TI was defined as the ratio of centerline vessel length divided by straight length (11). The TI values were measured twice for bilateral ICAs separately, and the mean values were recorded. When the TI values of bilateral ICAs were not consistent, lower TI value was chosen for further analysis.

### MRI Examination and Analysis

Magnetic resonance imaging examinations were performed using a 3.0T MRI system (MAGNETOM Skyra 3.0T, Siemens, Amberg, Germany). The parameters were as follows: (i) TR/TE = 4,730/72 ms, slice thickness = 4 mm, and FOV = 220 × 220 mm for T2WI; (ii) TR/TE = 7,500/81 ms, slice thickness = 4 mm, and FOV = 220 × 220 mm for FLAIR imaging; and (iii) TR/TE = 5,120/62 ms, slice thickness = 4 mm, FOV = 220 × 220 mm; (iv) three directions of diffusion gradient and two *b*-values (0 and 1,000 mm^2^/s) for DWI; and (v) TR/TE = 28/20 ms; slice thickness = 1 mm; and FOV = 220 × 220 mm for SWI. No contrast agent was administered (12).

The definition of SVD neuroimaging markers were as follows. Periventricular and deep WMHs were both coded on the basis of the Fazekas scale (grades 0–3), by using FLAIR and T2WI (12, 13). The definition of CMBs was homogeneous, small (<5 mm), round foci in the basal ganglia, white matter, cortico-subcortical junction, brainstem, or cerebellum, differentiated from mineral depositions in the globi pallidi and vessel flow voids, with hypointensity on gradient-echo images (12, 13). Lacunes were defined as round or ovoid lesions with a small (3–20 mm) diameter in the basal ganglia, internal capsule, centrum semiovale, or brainstem, with cerebrospinal fluid intensity on T2WI and FLAIR, generally with a high signal intense rim on FLAIR and no hyperintensity on DWI (12, 13). Enlarged perivascular spaces were defined as punctate or linear, small (<3 mm) hyperintensities in the basal ganglia or centrum semiovale on T2WI, and they were coded on the basis of a validated semiquantitative scale (grades 0–4) (12–14). In this study, we only counted EPVSs and CMBs in the basal ganglia region because the lesions in this region seemed associated with sporadic SVD specifically (13). There were two experienced neurologists, blinded to the patient's clinical information and the patient's CTA results, who independently reviewed the MRI images. They detected the presence of WMHs, CMBs, lacunes, and EPVSs according to the above diagnostic criteria. In the patients with acute infarct (based on DWI), these features were observed outside the acute infarct area. The patients with any of the MRI markers were classified into the positive case group. Furthermore, WMHs and EPVSs were divided into three subgroups according to the severity, respectively (15). Any disagreement was resolved by consensus.

### Clinical and Laboratory Information

Data on the traditional vascular risk factors (12, 16) and previous strokes in the patients were collected. Hypertension was defined as a resting systolic/diastolic blood pressure ≥140/90 mm Hg in repeated measurements or use of antihypertensive agents. Diabetes mellitus was diagnosed as present in the case of a fasting blood glucose level of ≥7.0 mmol/L or use of a hypoglycemic agent. Hyperlipidemia was defined as a low-density lipoprotein cholesterol level of ≥4.1 mmol/L, a total cholesterol level of ≥6.2 mmol/L, or use of an antilipemic agent. Previous stroke was diagnosed when a patient had a previous stroke-like symptom or an ischemic lesion was detected on brain imaging; transient ischemic attacks (TIAs) were excluded. Smokers were defined as those who smoked within 1 year before the stroke or had a current smoking habit. Atrial fibrillation was defined by an irregularly spaced QRS complex without a discrete P wave in electrocardiography. Coronary artery disease was diagnosed as a history of unstable angina, myocardial infarction, or occlusive disease of the coronary artery confirmed on angiography.

### Statistical Analysis

Student's *t*-test test was used to analyze continuous data. Univariate analysis was performed using the χ^2^-test for categorical data. The median TI value of ICA was compared among the subgroups with different grades of WMHs and EPVSs using the Kruskal–Wallis test. TI of ICA was compared between the subgroups with/without CMBs and lacunes using the Mann–Whitney *U*-test. Spearman rank correlation test was used to analyze the correlation between the TI value and each MRI marker of SVD, including WMHs, EPVSs, CMBs, and lacunes. Multivariate analysis was performed using logistic regression to investigate the association of ICA tortuosity with each of the SVD MRI markers. The multivariate analysis was adjusted for age, sex, diabetes mellitus, hypertension, hyperlipidemia, current smoking, previous stroke, prior coronary artery disease, atrial fibrillation, and three other SVD MRI markers (when one of the four neuroimaging markers was analyzed). The results were expressed as crude/adjusted odds ratios (ORs) and their 95% confidence intervals (CIs). The interobserver variability between the two readers was assessed on the basis of the kappa values. A value of *P* < 0.05 was considered significant. The statistical package SPSS 17.0 (IBM Corp., Armonk, NY, USA) was used for the analysis.

## Results

The interobserver agreement was found to be acceptable. The values for the presence of CMBs, EPVSs, WMHs, and lacunes were 0.936, 0.928, 0.912, and 0.909, respectively (all *P* < 0.001).

**Figure 2:**
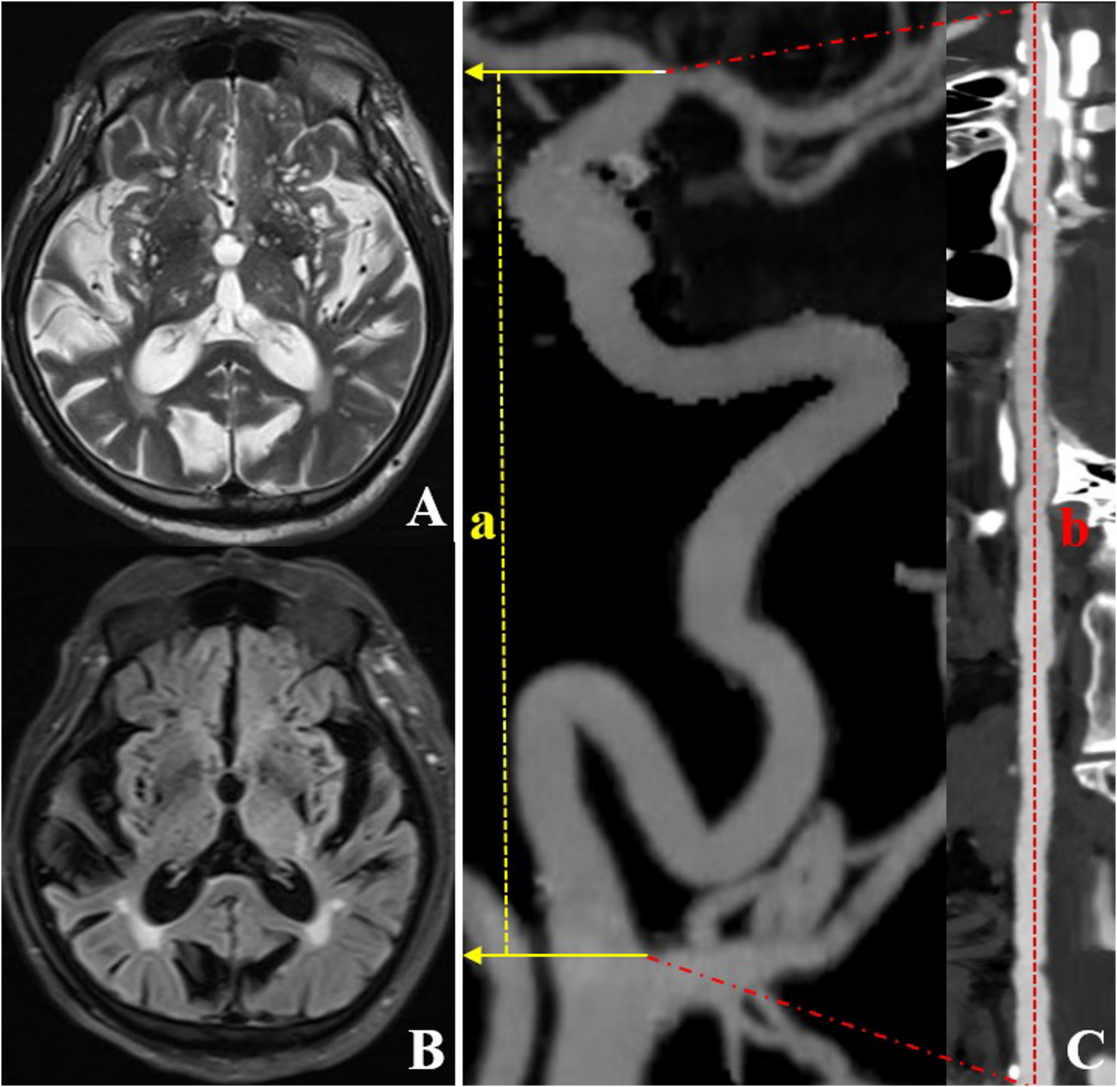
CTA and MRI images of an individual (a 72-year-old woman) with left ICA TI equal to 2.64. T2WI **(A)** and FLAIR **(B)** showed a grade 4 EPVS in the basal ganglia region. CTA with MIP **(C)** shows severe tortuosity of the left ICA. The dotted line a represents the straight length of the ICA, and the dotted line b represents the centerline vessel length of ICA reconstructed by using curved planar reformat. CTA, computed tomography angiography; EPVS, enlarged perivascular spaces; ICA, internal carotid artery; MIP, maximum intensity projection; MRI, magnetic resonance imaging; TI, tortuosity index.

**Figure 3:**
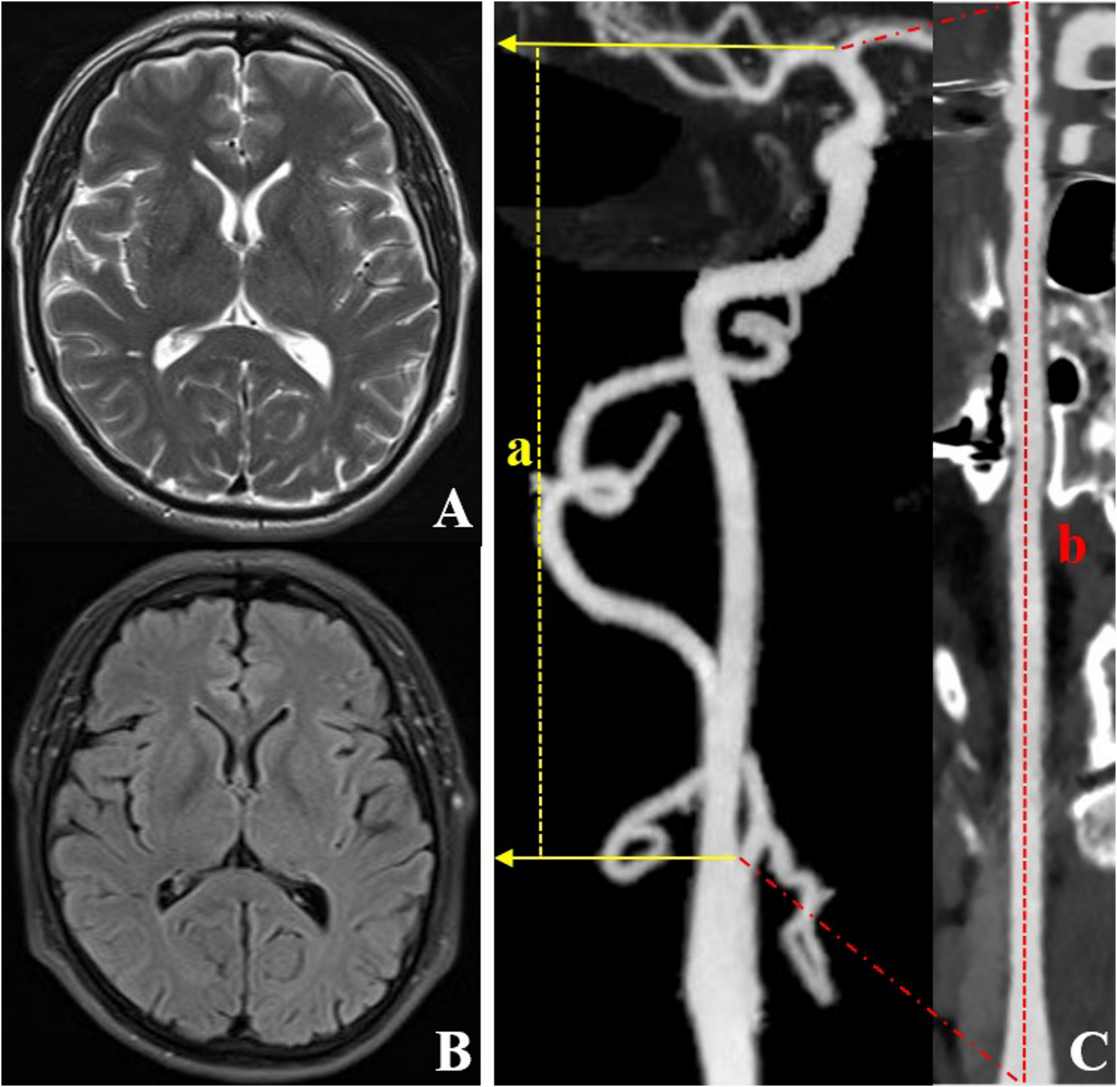
CTA and MRI images of an individual (a 59-year-old male) with right ICA TI equal to 1.33. T2WI **(A)** and FLAIR **(B)** show a grade 0 EPVS in the basal ganglia region. CTA with MIP **(C)** shows the lower tortuosity of the right ICA. The dotted line a represents the straight length of the ICA, and the dotted line b represents the centerline vessel length of ICA reconstructed by using a curved planar reformat. CTA, computed tomography angiography; EPVS, enlarged perivascular spaces; ICA, internal carotid artery; MIP, maximum intensity projection; MRI, magnetic resonance imaging; TI, tortuosity index.

**Figure 4:**
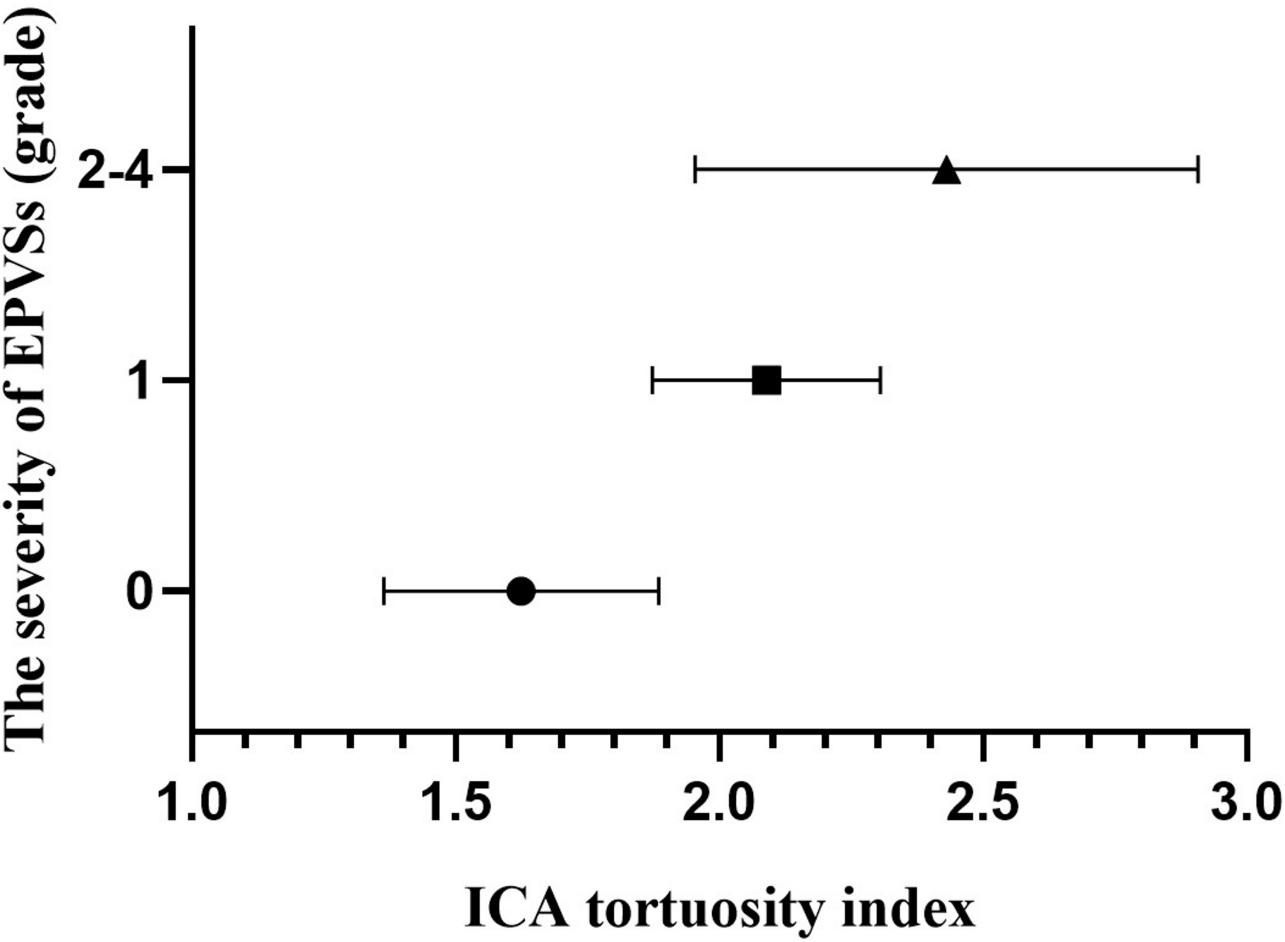
Column plot with mean and standard deviation values. ICA tortuosity is positively associated with the severity of EPVSs. EPVSs, enlarged perivascular spaces; ICA, internal carotid artery.

The baseline characteristics of the study population (*n* = 1,264) are shown in Table 1. Patient age and prevalence of hypertension, diabetes mellitus, current smoking, and previous stroke significantly differed between patients with and without SVD MRI spectrum (*P* < 0.001, respectively; Table 1). White matter hyperintensities were found in 47.4% of the subjects, lacunes in 47.3%, CMBs in 32.8%, and EPVSs in 27.0%. Univariate analysis showed that the ICA TI value was positively associated with the presence of each SVD marker (all *P* < 0.001; Figures 2, 3). The correlation coefficients (*r*_*s*_) were 0.57, 0.42, 0.30, and 0.26 for EPVSs, WMHs, CMBs, and lacunes, respectively (Table 2). The crude ORs of the ICA TI were 1.59 (95% CI 1.53–1.66, *P* < 0.001) for EPVS grade 1, 2.24 (95% CI 2.12–2.37, *P* < 0.001) for EPVS grades 2–4, 1.18 (95% CI 1.13–1.23, *P* < 0.001) for WMH grades 1–2, 1.62 (95% CI 1.53–1.71, *P* < 0.001) for WMH grade 3, 1.23 (95% CI 1.18–1.28, *P* < 0.001) for lacunes, and 1.31 (95% CI 1.25–1.37, *P* < 0.001) for CMB. After adjustment for age, sex, diabetes mellitus, hypertension, hyperlipidemia, current smoking, previous stroke, prior coronary artery disease, atrial fibrillation, and three other SVD MRI markers (when one of the four neuroimaging markers was analyzed), the adjusted ORs of the ICA TI were 1.52 (95% CI 1.44–1.60, *P* < 0.001) for EPVS grade 1, 2.05 (95% CI 1.93–2.18, *p* < 0.001) for EPVS grades 2–4, 1.01 (95% CI 0.97–1.05, *P* = 0.614) for WMH grades 1–2, 1.09 (95% CI 1.03–1.15, *P* = 0.004) for WMH grade 3, 0.97 (95% CI 0.94–1.01, *P* = 0.161) for lacunes, and 0.98 (95% CI 0.93–1.02, *P* = 0.233) for CMB (Table 3 and Figure 4).

**Table 1:** Baseline characteristics of subjects with and without SVD magnetic resonance imaging (MRI) markers.

**The presence of SVD neuroimaging**	**–(*n* = 464)**	**+(*n* = 800)**	***P*-value**
	***n* (%)**	***n* (%)**	
**Demographic data**			
Age, years*	59.21 ± 10.53	65.33 ± 10.54	<0.001
Male	206 (36.3)	361 (63.7)	0.802
Female	258 (37.0)	439 (63.0)	
**Risk factors**			
Hypertension*	108 (19.1)	456 (80.9)	<0.001
Diabetes mellitus*	91 (26.0)	259 (74.0)	<0.001
Hyperlipidemia	53 (30.5)	121 (69.5)	0.066
Previous stroke*	44 (16.4)	224 (83.6)	<0.001
Current smoking*	69 (25.3)	204 (74.7)	<0.001
Atrial fibrillation	36 (28.8)	89 (71.2)	0.053
Prior coronary artery disease	81 (32.3)	170 (67.7)	0.103
Tortuosity index*	1.72 ± 0.33	1.81 ± 0.42	<0.001

*SVD, small vessel disease*.

Each baseline characteristic was compared between subjects with and without SVD MRI markers. *Significant at P < 0.05.

**Table 2:** Comparison of ICA tortuosity according to the MRI markers of SVD.

**SVD**		***n* (%)**	**TI mean value ± SD**	***P*-value**	***r_***s***_***
WMHs	Grade 0	665 (52.6)	1.65 ± 0.30	<0.001	0.42
	Grades 1–2	379 (30.0)	1.81 ± 0.38		
	Grade 3	220 (17.4)	2.13 ± 0.40		
EPVSs	Grade 0	923 (73.0)	1.62 ± 0.26	<0.001	0.57
	Grade 1	230 (18.2)	2.09 ± 0.22		
	Grades 2–4	111 (8.8)	2.43 ± 0.48		
Lacunes	–	666 (52.7)	1.68 ± 0.32	<0.001	0.26
	+	598 (47.3)	1.89 ± 0.42		
CMBs	–	850 (67.2)	1.69 ± 0.34	<0.001	0.30
	+	414 (32.8)	1.96 ± 0.42		

*CMBs, cerebral microbleeds; EPVSs, enlarge perivascular spaces; ICA, internal carotid artery; r_s_, correlation coefficient; SVD, small vessel disease; TI, tortuosity index; WMHs, white matter hyperintensities*.

*The TI of ICA was compared according to the presence of each SVD marker, including WMHs, EPVSs, lacunes, and CMBs according to the severity*.

**Table 3:** ICA tortuosity in relation to the different MRI markers of SVD.

**SVD**	**Crude OR (95% CI)**	***P*-value**	**Adjusted OR (95% CI)**	***P*-value**
EPVS (grade 1)	1.59 (1.53–1.66)	<0.001	1.52 (1.44–1.60)	<0.001
EPVS (grades 2–4)	2.24 (2.12–2.37)	<0.001	2.05 (1.93–2.18)	<0.001
WMH (grades 1–2)	1.18 (1.13–1.23)	<0.001	1.01 (0.97–1.05)	0.614
WMH (grade 3)	1.62 (1.53–1.71)	<0.001	1.09 (1.03–1.15)	0.004
Lacunar	1.23 (1.18–1.28)	<0.001	0.97 (0.94–1.01)	0.161
CMB	1.31 (1.25–1.37)	<0.001	0.98 (0.93–1.02)	0.233

*CMB, cerebral microbleed; EPVS, enlarge perivascular space; ICA, internal carotid artery; OR, odds ratio (per unit increase in the explanatory variable); SVD, small vessel disease; WMH, white matter hyperintensity*.

*Adjusted OR was calculated using a multivariable analysis involving the following variables: age, sex, hypertension, diabetes mellitus, hyperlipidemia, previous stroke, current smoking, atrial fibrillation, prior coronary artery disease, and three other MRI markers of SVD (when one of the four neuroimaging markers was analyzed)*.

## Discussion

In the current study, the TI of ICA was found to be higher in patients with the neuroimaging of SVD. After adjustment for the confounding factors, ICA tortuosity was associated with MRI-defined markers of SVD, including EVPSs and high-grade WMHs; furthermore, ICA tortuosity was positively correlated with EPVS severity.

Our findings suggested that ICA tortuosity was significantly correlated with EPVSs. In the previous studies, arterial tortuosity was reported to correlate with the neuroimaging of SVD (17–20), while only a few studies have addressed EPVSs. Pico et al. (7) found that patients with ischemic stroke and intracranial arterial dolichoectasia (IADE) have a higher prevalence of EPVSs. However, the diagnosis for IADE was made by consensus based on the visual impression and without diagnosis criteria. Moreover, the study only involved three imaging markers of SVD (WMHs, EPVSs, and lacunes). In the present study, we used a TI value to evaluate the severity of ICA tortuosity because it was a relatively straightforward approach in the absence of diagnostic criteria for anterior circulation vessel tortuosity. Furthermore, we investigated the correlation between ICA tortuosity and four imaging markers of SVD, since several MRI features are considered to often co-occur and might be confounding factors to each other (15). We assumed that there were several possible explanations for our findings. Blood vessel tortuosity was a systemic problem and could also affect small vessels (1). Brain small vessel tortuosity could lead to stiffer arteries (21) and transmit greater pulsatility to the brain parenchyma (22), which might lead to enlargement of perivascular spaces. Additionally, the tortuous cerebral arterioles slightly extend and wind on themselves, while pushing back into the perivascular space. Simultaneously or subsequently, there is a loss of brain parenchyma around the tortuous vessel, resulting in an EPVS to permit further tortuosity of the blood vessel (18). This might be a long and dynamic process, so we also found that ICA tortuosity was positively correlated with EPVS severity. These findings are interesting because the pathophysiology of EPVS is unclear, and these results need to be confirmed in further studies.

Additionally, we found that ICA tortuosity was only correlated with the high-grade WMHs (grade 3) after adjustment for potential confounding factors. Our results were not completely consistent with the results of previous studies. These inconsistencies may be due to differences in study cohort and methodology. Some studies established an association between IADE and WMHs (7–9). However, one was a brain-autopsy study concerning fatal stroke patients (9), and others concerned posterior circulation arterial tortuosity (7, 8). Some studies also reported a higher prevalence of lacunar infarctions in the patients with IADE and stroke (23). Park et al. (24) found that vertebrobasilar dolichoectasia in patients with TIA or stroke was associated with CMBs. However, they investigated posterior circulation arterial tortuosity in stroke patients, and the sample size was rather small. In the present study, the individuals were suspected of having cerebral vessel diseases, not only the patients with stroke. Individuals who had large area infarction, hemorrhagic stroke, and ipsilateral artery stenosis >50% were also excluded. Furthermore, this study only concerned ICA tortuosity. Therefore, our findings might be somewhat different from those reported in other studies. Further studies including anterior and posterior circulation arterial tortuosity are needed to confirm the results.

Previous studies mainly analyzed the relationship between IADE and SVD; however, there are many tortuosity cases that do not involve dilatation. In addition, Smoker's criteria provided a relatively consistent means to diagnose posterior circulation IADE (25); however, there were no diagnostic criteria for anterior circulation IADE (2). Several possible measures of tortuosity have been suggested, but none of them have obtained universal acceptance. Furthermore, anterior and posterior circulation arterial tortuosity does not coexist in many cases, which may due to different structures surrounding the vessels. Therefore, in the present study, we only measured the ICA tortuosity and used TI to evaluate the severity of tortuosity because of its ease of computation and conceptual simplicity.

This study has several limitations. First, the severity of atherosclerosis is different among individuals; however, almost 20% of IADE patients do not have traditional cardiovascular risk factors, suggesting that IADE can occur without atherosclerosis (25). Second, our controls did not represent “healthy” controls because completely healthy populations cannot undergo CTA; we selected the less severe side of the ICA for analysis when the degree of tortuosity of both ICAs was not consistent. However, both conditions may lead to an underestimation of the association between the SVD severity and TI value. Third, it is difficult to conclude the grading standard for ICA tortuosity in this study because we only analyzed the patients with SVD MR features, and the sample size of the patients with EPVSs was relatively small. Last, the current study had a retrospective design, which was a substantial limitation.

In conclusion, the TI of ICA was higher in patients with neuroimaging of SVD. Internal carotid arteries tortuosity was associated with SVD MRI-defined markers, including EVPSs and high-grade WMHs. Internal carotid arteries tortuosity was positively correlated with EPVS severity. Arterial tortuosity might be a risk factor for SVD with implications for understanding the pathophysiology of SVD. This finding may have potential clinical significance for identifying individuals with suspected SVD and evaluating disease severity.

## Data Availability Statement

The raw data supporting the conclusions of this article will be made available by the authors, without undue reservation.

## Ethics Statement

The studies involving human participants were reviewed and approved by the Shanghai Jiao Tong University Affiliated Sixth People's Hospital institutional review board. The patients/participants provided their written informed consent to participate in this study.

## Author Contributions

Y-HL and Y-CC contributed conception and design of the study. Y-CC, R-HQ, and X-FS organized the database. X-EW and JL performed the statistical analysis. Y-CC wrote the first draft of the manuscript. Y-HL, Y-CC, X-EW, and JL wrote sections of the manuscript. All authors contributed to manuscript revision, read and approved the submitted version.

## Conflict of Interest

The authors declare that the research was conducted in the absence of any commercial or financial relationships that could be construed as a potential conflict of interest.
